# Phase Difference Optimization of Dual-Wavelength Excitation for the CW-Photoacoustic-Based Noninvasive and Selective Investigation of Aqueous Solutions of Glucose

**DOI:** 10.3390/s150716358

**Published:** 2015-07-07

**Authors:** Serge Camou

**Affiliations:** NTT Device Technology Laboratories, NTT Corporation, Atsugi 243-0198, Japan; E-Mail: camou.serge@lab.ntt.co.jp; Tel.: +81-46-240-2319; Fax: +81-46-240-2936

**Keywords:** CW-photoacoustic, noninvasive monitoring, glucose, differential measurement, dual wavelength excitation

## Abstract

Towards the noninvasive and continuous monitoring of blood glucose levels, we chose the continuous-wave photoacoustic (CW-PA) technique and developed the optical power balance shift (OPBS) method. However, operating with optical wavelengths in the near-infrared (NIR) region ensures deep penetration inside human soft-tissue, but also leads to two serious issues: strong background level noise from water molecules in this wavelength range and small differences between the absorbance spectra of diluted compounds. To resolve them, the OPBS method relies on simultaneous optical excitation at two wavelengths for differential measurements. However, the first validation *in vitro* with calibrated aqueous solutions of glucose and albumin revealed strong dependence on the phase difference between the two lights sources. In this paper, we report a systematic investigation of this parameter, from PA-based measurements over a wide range of phase differences and an extensive characterization in the frequency domain. The process of maintaining the phase quadrature of the two optical signals is demonstrated in real time through an analysis of the PA signal and therefore does not require any additional equipment. Finally, a comparison of aqueous glucose solution characterizations at high concentration levels with the two methods was performed and consistent results were obtained.

## 1. Introduction

Diabetes mellitus [1,2], often referred to as diabetes, comprises a group of common metabolic disorders that result in loose control of the blood glucose level (BGL) and can lead to hyperglycemia (elevated blood sugar levels) with multiple complications [3,4]. Maintaining the BGL within the range normally found in a healthy person is the basic way to prevent the negative impacts of diabetes on the patient’s health. Every time the BGL exceeds the normal limit [5,6], adequate actions, depending on the type of diabetes, should be taken to restore the level into the admissible range. However, efforts and actions aiming at lower BGL also expose patients to hypoglycemia, which is associated with BGL lower than 60 mg/dL [7]. Accurate, on-site and real time detection of BGL represents the initial and essential step for adequate decision-making. Over the past decades, several commercially available sensors based on blood analysis have emerged. Due to their compact size, low cost, good accuracy, and fast response [7,8,9], these sensors have not only rapidly gained worldwide popularity, but have also provided huge benefits to the diabetic population. However, despite significant efforts to minimize the volume of investigated blood samples and reduce the discomfort associated with finger-pricking, these sensors are still invasive by design and are therefore not suitable for continuous monitoring, which is a major corner-stone for optimal BGL control [10,11].

To achieve the ultimate goal, minimally invasive (MI) techniques have emerged. They are characterized by the fact that the head of the sensor (reduced to its minimum and inserted subcutaneously) is in direct contact with body fluids while the signal processing is performed outside the body. This approach reduces the invasive character and enables continuous monitoring over periods that can extend over several days [12,13,14]. However, despite the reduced size and minimum discomfort, the implantation of such a device in a human body poses the problem of biofouling [15]. As a result, commercially available products claim to provide accurate reading for periods no longer than three days, though researchers are currently developing technologies for long-term readings of up to several months [16,17].

Undoubtedly noninvasive methods, currently less advanced than the MI approach, remain the ultimate and perfect solutions [18,19]. However, great care should be paid to two particular aspects: the sensitivity and the selectivity of the measurements. Among potential alternative candidate techniques [20,21,22,23,24,25,26,27,28], photoacoustic (PA) methods (pulse and continuous wave (CW)) do not exhibit the limitations due to scattering inherent to purely optical-based methodologies and therefore provide an interesting alternative when dealing with human tissues [23,24,25,26,28]. For these reasons, we chose the CW-PA methodology and we have already developed two methods called “frequency shift” (FS) [29] and “optical power balance shift” (OPBS) [30,31]. While the FS approach is well understood and provides a robust principle that does not require any further optimization, the OPBS one relies on the use of two light sources operating at different optical wavelengths. The concept of dual-wavelength excitation was first proposed by Niessner *et al.* [32] several years ago as an efficient way to overcome strong absorption by water molecules in the near infrared (NIR) region when wavelengths are chosen appropriately. First designed for purely optical measurements, the concept was further extended to the PA technique by the same authors [33]. However, several problems were reported, notably concerning the phase difference adjustment between the two optical signals.

In this work, we first investigated the influence of the phase difference between the two channels. The results demonstrate a considerable impact on the response characteristics, as similarly reported in [33]. To further validate the concept, we also conducted an analysis of the PA signal in the frequency domain based on fast-Fourier transform, which further confirmed the trend. Finally, we performed measurements of aqueous glucose solution using the two protocols and compared the results.

## 2. Experimental Section

### 2.1. OPBS Method

Whenever an absorbing medium is illuminated by two optical beams with independent output power levels, both amplitude-modulated (for example with a square wave) at the same frequency, but of opposite phase (*i.e.*, 180 degree-phase shift between the two signals), the equation of the generated acoustic pressure [34] can be rewritten as

(1) $$S\propto (\alpha_{1}P_{1}-\alpha_{2}P_{2})$$

where *S* is the acoustic pressure (linearly proportional to the output voltage from the transducer), α the optical absorption coefficient, and *P* the optical power. The subscripts 1 and 2 are related to optical wavelengths 1 and 2, respectively.

In Equation (1), acoustic pressure *S* also depends on several other parameters involved in the generation of acoustic waves such as acoustic velocity and heat capacity. However, the OPBS protocol relies on the adjustment of optical powers *P*_1_ and *P*_2_ in order to minimize or cancel out the generation of the acoustic wave in the propagation medium. As a result, it is possible to neglect the influence of all of the other parameters involved and exclusively focus on the difference in α*P* at the two optical wavelengths chosen.

The optical absorption at one wavelength depends on the concentration of diluted compound according to the following linear equation:

(2) $$\alpha (t)=\alpha (t_{0})+\Delta C_{A}\cdot \delta \alpha_{A}+\Delta C_{B}\cdot \delta \alpha_{B}\;with\;\alpha (t_{0})=\alpha_{water}+{C_{A}|}_{t_{o}}\cdot \delta \alpha_{A}+{C_{B}|}_{t_{o}}\cdot \delta \alpha_{B}$$

with *A* and *B* the diluted compounds with their corresponding concentration *C* and fractional absorption coefficient δα. Any change in the concentration of compound A or B then results in a change in the absorption according to Equation (2). The OPBS basic concept then consists of manually adjusting the optical power parameters *P* in Equation (1) through the laser diode (LD) driving voltage (DV) in order to remain at the amplitude minimum/phase inflection point. Moreover, it has been shown that the optical power change required to minimize the acoustic signal, *i.e.*, to compensate for the absorbance change, is linearly proportional to the concentration change [31] and can therefore be used to monitor the concentration change of the sample liquid.

### 2.2. Experimental Setup

**Figure 1:**
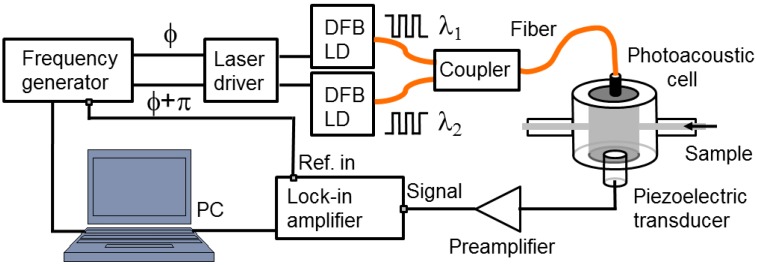
Schematic view of the experimental setup for OPBS-based measurements.

Figure 1 is a schematic view of the experimental setup used to perform the PA-based study of aqueous solutions of glucose. The frequency generator (FG) (WF1948, NF, Yokohama, Japan) generates a square-wave voltage signal that triggers the SR844RF lock-in amplifier (Stanford Research System, Sunnyvale, CA, USA) and drives the two distributed feedback LDs (NTT Electronics, Yokohama, Japan) at the same modulation frequency but in the opposing phase, while the voltage levels can be adjusted independently. The two optical signals modulated in intensity are then combined through a coupler and sent to the measuring cell through a single-mode optical fiber. On the other side of the cell, the acoustic pressure sensed by the transducer (Acoustic Emission R-CAST M-204A, Fujicera, Kyoto, Japan) is first pre-amplified (Pre-Amp Unit A1002, Fujicera, Japan) before being fed into the lock-in amplifier. A computer controls all components via a MATLAB GUI interface (MATLAB R2012b, Natick, USA). This experimental system enables amplitude and phase measurements at any frequency within the bandwidth of the transducer, *i.e.*, between 300 and 600 kHz.

The custom-made detection cell [35] is a resonant cavity with a cylindrical shape. The length of the cavity can be adjusted continuously by rotating the holder of the optical fiber; the optical fiber tip and acoustic transducer aligned along the symmetry axis are facing each other. Inlet and outlet ports enable us to replace the sample solution while continuously capturing CW-PA signals.

### 2.3. Glucose Solution Preparation

Solutions were prepared by dissolving dried pure glucose (d-(+)-Glucose, G5767, Sigma-Aldrich Japan, Tokyo, Japan) in deionized water. Glucose is highly soluble in water, which permits easy preparation of solutions for concentrations in the range of several grams per deciliter.

### 2.4. Experimental Sequence

In the CW-PA method, the generated standing acoustic waves resonate at certain frequencies depending on the boundary dimensions and the characteristics of the propagation medium. Performing measurements at the resonance frequencies strongly enhances the signal levels for more sensitive data collection. However, even if the boundary dimensions can be assumed to be constant, the propagation medium is sensitive to many parameters (such as temperature and compound concentration), whose change results in a shift of the resonance frequency. In order to compensate for any frequency shift and remain at resonance at all times, the modulation frequency of the LDs is adjusted every time a sample solution is transferred to the detection cell with a different concentration. This protocol, which can also provide measurements of liquid sample characteristics, has already been described in detail elsewhere [29].

**Figure 2:**
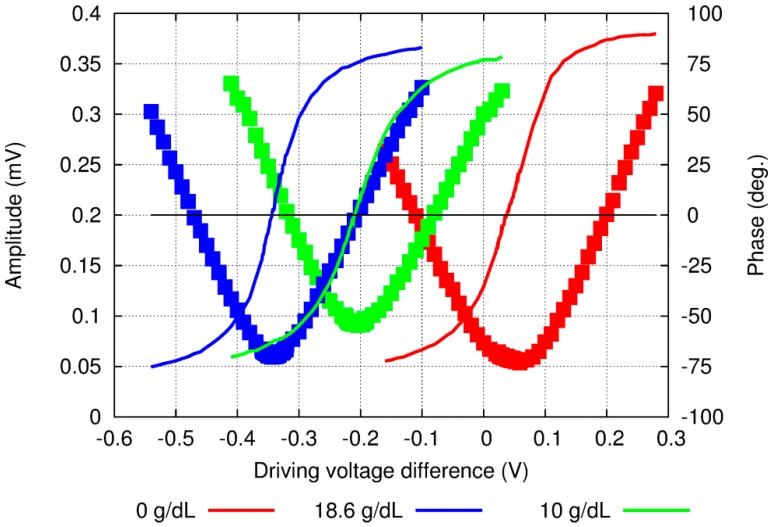
Amplitude (dots) and phase (lines) raw experimental results when the DV was scanned around the 0-phase value at several concentrations of aqueous solution of glucose.

The OPBS technique is a relative method: It requires the availability of a reference with a known concentration. Figure 2 shows one experimental set of data with water as the reference sample solution; however, any liquid may be used as long as the concentration is known.

The first step consists of sweeping the DV of the two LDs sequentially with a calibrated sample solution. From a practical point of view, the LDs’ DV should not exceed a certain value in order to (i) remain within the linear regime of the LDs and (ii) not to exceed the output optical power required to alter the solution through irreversible phenomena such as photo-ablation and vaporization [36]. The horizontal axis in Figure 2 is then calculated as the difference between the two DVs, *i.e.*, DV_1_–DV_2_, where subscripts 1 and 2 refers to wavelengths 1 and 2.

A typical response, including the amplitude and phase signals, then exhibits a minimum on the amplitude and an inflection point on the phase at the same difference between DVs (Figure 2). When the signals at the two wavelengths are strongly unbalanced, the resulting acoustic signal exhibits a high amplitude level as well as the phase of the main contributing signal, *i.e.*, −90° or 90°, depending on which side is being considered. Furthermore, when the two signals are well balanced, the amplitude signal is minimal while the phase exhibits an inflexion point at 0°. Next, a sample solution including the compound of interest is introduced into the measuring cell. Because of the difference in the optical absorption at the two wavelengths used, the signal at the 0-reference point corresponds neither to the amplitude minimum nor to the phase inflexion point any longer. A new scan over the DV difference reveals a response shifted by a quantity δDV, which can be easily obtained from the amplitude or the phase signal. However, because of the round-shape of the amplitude minimum and inherent low signal-to-noise ratio, the phase signal is preferably used. The measured δDV shift is then compensating for the unbalanced signals due to the difference in the optical absorption at the two wavelengths, which is itself proportional to the variations in concentration. This δDV parameter provides an efficient tool to measure any change in compound concentration. Since this process can be performed consecutively, it enables continuous monitoring of the sample’s solution.

## 3. Results and Discussion

### 3.1. PA Signal for Wide Range of Phase Differences and Driving Voltages

The proposed OPBS method was tested with aqueous solution of glucose at high concentration levels and optical wavelengths of 1382 and 1610 nm. These two wavelengths were selected because of the similar absorption coefficients of water, while the fractional absorptions of glucose are distinct and of opposite signs [37]. Figure 2 shows the raw results around the amplitude minima/phase inflexion points. However, these results clearly show two issues: (i) it is not possible to decrease the signal amplitude levels below 50 microvolts and (ii) the slope of the phase signal, despite remaining locally linear around the inflection point, exhibits different values. Furthermore, when repeating the experiments, different behaviors, *i.e.*, a steeper slope or lower amplitude signal levels at the minimum, were also obtained.

**Figure 3:**
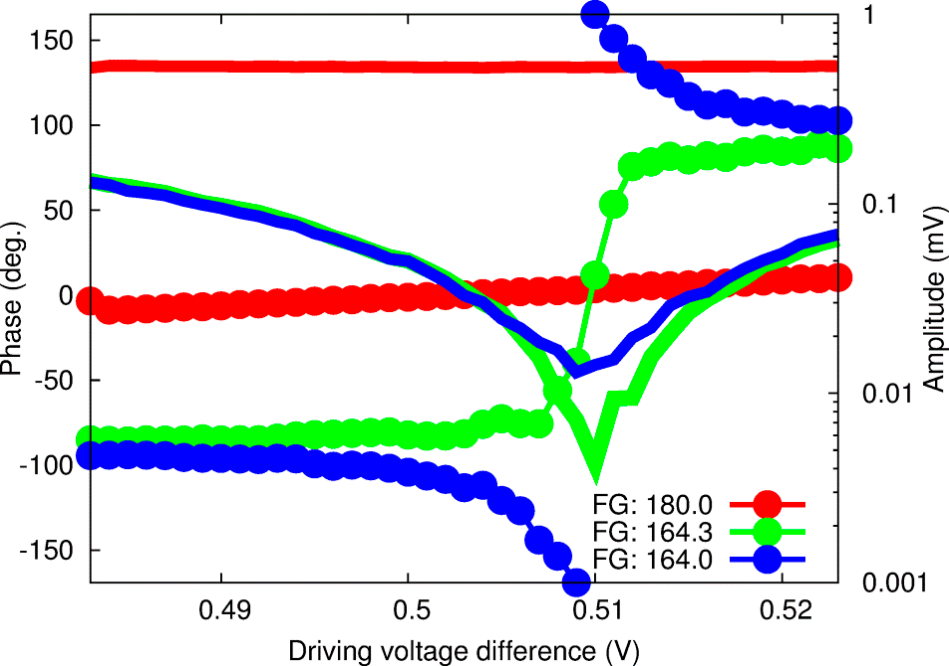
OPBS-based amplitude (plain lines) and phase (dotted lines) response for pure water at three phase differences between the two optical wavelengths.

**Figure 4:**
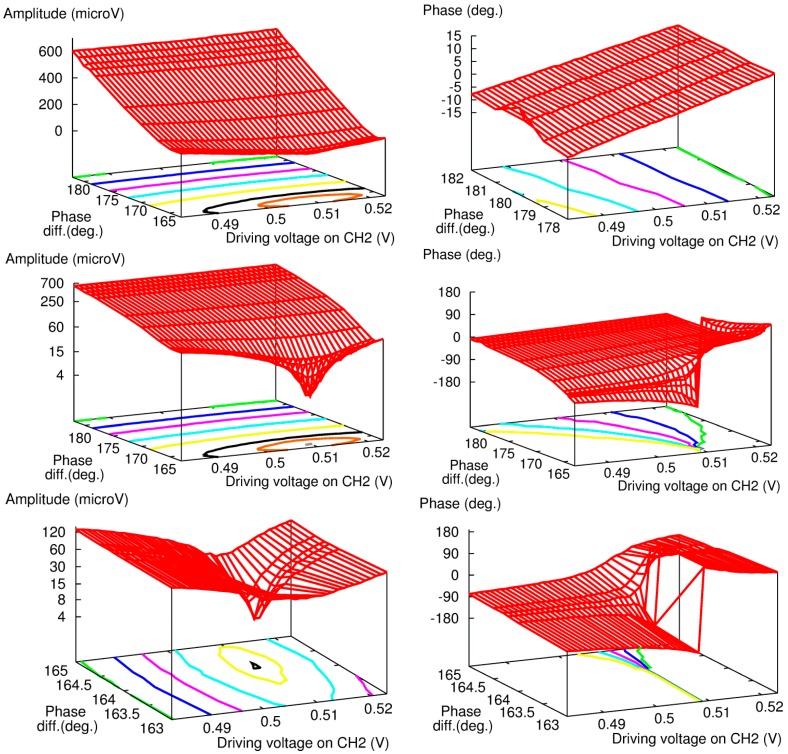
Systematic measurement of amplitude (**Left**) and phase (**Right**) of PA signal over a wide range of DV and phase difference at FG channel 2.

To further investigate these issues, we then added the phase difference between the two channels as a parameter. Figure 3 shows the results for three different phase difference values, where “FG: 180.0” means a 180° phase difference at the frequency generator and “FG: 164.3” and “FG: 164.0” correspond to the minimum amplitude signal and the rotating phase around the amplitude minimum value, respectively. Results of a systematic investigation along the two parameters—the phase difference and the driving voltage—are shown in Figure 4.

At the phase difference of 164.3°, the amplitude signal is just a few microvolts high (~4 MV), a level more consistent with our first assumption of no generation of an acoustic wave. Moreover, the phase signal exhibits a steep change around this point, almost similar to a step function. Repeating similar experiments several times also revealed that this phase difference drifts with time. It is then necessary to readjust it as often as possible in order to minimize the amplitude signal to only a few microvolts. This characteristic can therefore explain the change in the slope or amplitude signals at the minima reported previously, since a constant phase difference of 180° at the FG was always used. The capacity to compensate this shift in real time is also an advantage compared to previously reported methods based on similar dual-wavelengths excitation, where only variations in amplitude signal, including shift due to any potential drift of the phase difference, were measured [32,33]. It should be noted that, at the present stage, it is impossible to identify with certainty the cause of this shift, which could come from any of the equipment between the FG and the fiber coupler, which includes the voltage/current converter and the light source.

### 3.2. Analysis in the Frequency Domain

**Figure 5:**
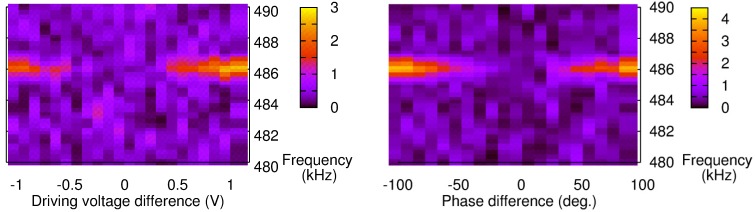
Fast Fourier Transform-based characterization of the PA signal with a Phase-locked loop operating at 486.2 kHz and for various DVs (**Left**) or phase differences (**Right**).

To confirm these findings, we then performed an analysis of the acoustic signal detected by the transducer in the frequency domain. At this stage, we monitored a response peak at a frequency of 486.2 kHz. We first made the adjustments along the phase difference and voltage based on minimizing the PA signal. Then, we replaced the lock-in amplifier with an oscilloscope and performed decomposition of the acoustic signal in the frequency domain. Figure 5 shows the results around the fundamental frequency when the phase difference or voltage was changed from the reference point. In the graph, the 0 abscissa corresponds to the balanced configuration (two signals balanced, so no emission of an acoustic wave). This point was determined on the basis of the previously described PA procedure and, as expected, there is no signal captured at the resonance frequency.

**Figure 6:**
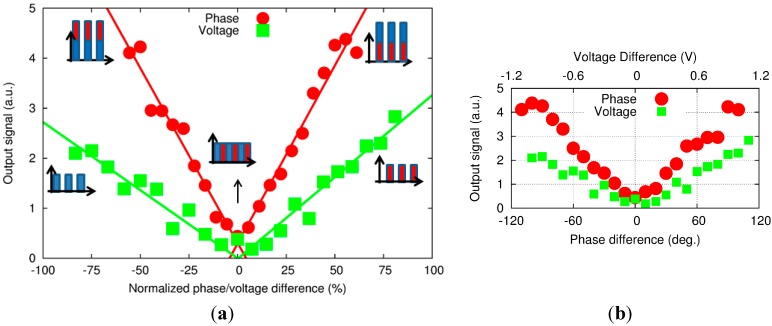
(**a**) PA amplitude signal *versus* normalized phase difference and voltage with (**b**) the corresponding raw data.

Then, changing the phase difference or DV of ch2 yields a contribution at the excitation frequency (Figure 5). The intensity, or height, of the peak also increases as we go further away from the 0-reference point. However, despite a similar trend in both results, the amplitude seems higher when the phase difference is changed. In order to compare the two phenomena, the raw data obtained from the peak height in the frequency domain (Figure 6b) were normalized. A 100% normalized phase difference corresponds to a 180° phase shift, while the equivalent in DV corresponds to a shift by the difference between DV at the balance level and the threshold voltage of the LDs. The results are shown in Figure 6.

The experimental results show that the PA signal is about 2.3 times more sensitive to the phase difference than DV. However, these results can be explained with the graph in Figure 6. With a −100% on the DV, the remaining acoustic signal is generated by only one signal wavelength. However, with a −100% on the phase difference, the two signals at the two wavelengths are at equal height (balanced) but superimposing on each other (in phase, or 0-phase difference) and thereby generating a square wave about twice as high as the single wavelength signal. As a result, the acoustic signal is doubled compared with the single wave excitation. Once again, the experiments yielded a factor of 2.3, which is consistent with the factor of 2 expected from theoretical consideration.

### 3.3. Measurements of Aqueous Glucose Solution

**Figure 7:**
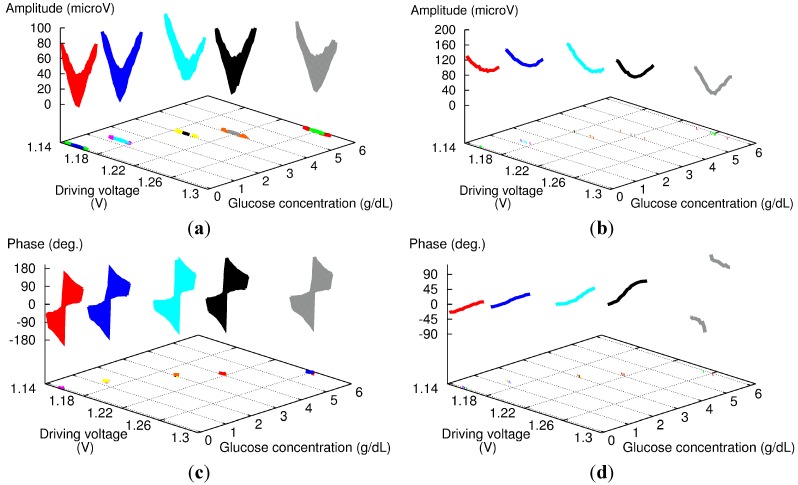
Experimental amplitude (**a**,**b**) and phase (**c**,**d**) results for 180-phase difference set at the fiber coupler (**a**,**c**) and FG (**b**,**d**) for aqueous glucose solutions with high concentration levels.

To estimate the benefit of including the phase difference adjustment in the measurement protocol, we measured an aqueous solution of glucose at a concentration of several grams per deciliter with and without the phase adjustment described above. The raw results are displayed in Figure 7, with the amplitude (top) and phase signal (bottom) for the protocol with the phase adjustment (left) and the one with the constant phase difference of 180° set at the FG (right). For both series, we performed measurements at several DVs and phase differences around the balanced point to assess the overall shape of the sensor response.

With the phase adjustment (left of Figure 7), both the amplitude and phase responses exhibit good reproducibility whatever the concentration: the amplitude shows a sharp V-shape response, and the phase response a 360° rotation of the phase. These results are consistent with those shown in Figure 3 with “FG: 164.3” and “FG: 164.0”.

However, the results without phase adjustment (right of Figure 7) show strong dependence *versus* glucose concentration. The amplitude signal always exhibits a round-shape response, but becomes more pronounced as the glucose concentration increases. The results for the phase signal are similar: as the glucose concentration increases, the slope of the phase increases, and the last measurement even features a 360° rotation of the phase. It should be noted that from our experience, the most important parameter here is more likely the time, not the change in glucose concentration. The measurement sequence started with pure water, and then the glucose concentration of sample solution was increased gradually to a maximum of 5.9 g/dL. Furthermore, we could obtain similar results without changing the composition of the sample solution just by repeating the same experiment several times over a day.

**Figure 8:**
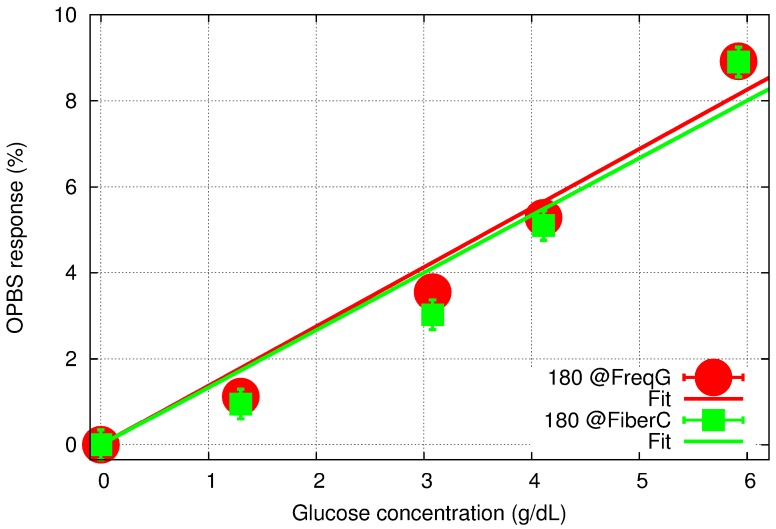
OPBS-based sensor response to aqueous glucose concentration for 180-phase difference set at the FG (red) and the fiber coupler (green), and their corresponding linear regression fitting.

Figure 8 summarizes the results with a comparison of the two responses. To estimate the measurement accuracy (error bars in Figure 8), a value of ±4 mV was used for all the data points. In the case of the 180° phase difference at the fiber coupler, this value includes a ±2 mV error due to the fact that measurements were performed in a region characterized by low signal levels and therefore by a low SNR for several points in the vicinity of the measured absolute minimum. Furthermore, it also accounts for the error in the phase difference, which can introduce an overall shift of the V-shape response as can be seen in Figure 3. In the case of the 180° phase difference at FG, the resolution is higher due to linear regression around the 0-phase point, but any error in phase difference still impacts the final measurement accuracy. As a result, the error of ±4 mV used in Figure 8 represents an overestimate of the actual resolution for both methods, but it provides valuable information for further comparison of the two sets of data.

First, the two sets of results are consistent and exhibit an overall linear tendency of a change in glucose concentration with the slope in the range of 1.33% to 1.38%/g/dL. However, the new method does not provide any improvement in the response linearity. The concentration range studied in this manuscript is several orders of magnitude higher than the expected levels in human tissues, which is typically in the range of 40 to 400 mg/dL. In order to extend the dynamic range of our sensor to these low concentration levels, further improvement of the measurement accuracy is needed. We believe that correcting the phase difference in real time is an important step forward towards this direction. However, the aqueous glucose measurements reported here also revealed that the limiting factor might lie elsewhere.

### 3.4. Remaining Issues

The proposed OPBS method differs from all other previously reported methods and its exact mechanism is not yet fully understood at this stage. With the steadily increasing number of experimental parameters, systematic optimization of the procedure is time consuming and future experiments will mainly focus on the following issues.

To explain the results in Figure 8, we should keep in mind that the proposed OPBS measurement remains an amplitude-based methodology. As a consequence, any instability in LD output can alter the reading of the sensor and be misinterpreted as a change in concentration. Therefore, it is of vital importance to monitor and compensate for any change in the output signal of the LDs in order to further extend the application of OPBS and eventually reach the glucose concentration range characteristic for diabetic patients. As a consequence, these issues should be investigated extensively before any discussion about the detection limit of the proposed method.

## 4. Conclusions

This contribution further explored the characteristics of the CW-PA-based OPBS protocol, particularly the dual-wavelength excitation for differential measurements. As reported in previous work, the phase difference between the two optical signals plays an important role, and we confirmed this with experimental validation based on PA measurements as well as characterization of the acoustic signal in the frequency domain. Taking this parameter into account allows us to minimize the amplitude signal to levels in the range of a few microvolts, with a phase response similar to a step function. Compared to other contributions using the same differential technique, our approach allows real-time adjustment of this parameter to keep the reference constant, a unique feature that should enhance the accuracy and reliability of the measurements. Characterization of aqueous glucose solutions at high concentration levels was also reported. The results show good consistency, but no improvement of the linearity could be obtained by using phase adjustment. In order to quantify the effect of the proposed phase adjustment, we need to resolve other experimental issues.

Despite a better understanding of the basic concept, the factors limiting the measurement accuracy remain unclear. Further work should focus on monitoring and compensating for any fluctuation of the light source output power in order to operate our sensor in the range consistent with glucose levels found in human tissues, *i.e.*, between 50 and 400 mg/dL, and with an accuracy of a few tens of milligrams per deciliter.
